# Qualitative analysis of stakeholder perspectives on engaging Latinx patients in kidney-related research

**DOI:** 10.1186/s12882-023-03128-y

**Published:** 2023-03-30

**Authors:** Flor Alvarado, Cynthia Delgado, Susanne B. Nicholas, Allison Jaure, Lilia Cervantes

**Affiliations:** 1 Section of Nephrology and Hypertension, Department of Medicine, Tulane University, 1430 Tulane Avenue #8545, New Orleans, LA 70112 USA; 2 Section of Nephrology, San Francisco VA Medical Center and University of California San Francisco, San Francisco, CA USA; 3 Division of Nephrology, Department of Medicine, David Geffen School of Medicine at University of California Los Angeles, Los Angeles, CA USA; 4 Centre for Kidney Research, Children’s Hospital at Westmead, Westmead, NSW Australia; 5 Sydney School of Public Health, University of Sydney, Sydney, Australia; 6 Department of Medicine, University of Colorado, Anschutz Medical Campus, Aurora, CO USA

**Keywords:** Barriers, Community-based participatory research, Cultural responsiveness, Disparities, Engagement, Health equity, Kidney disease, Research participation

## Abstract

**Background:**

Latinx individuals are disproportionally burdened by kidney diseases compared to non-Latinx White individuals and are underrepresented in kidney-related research. We aimed to describe stakeholder perspectives on Latinx patient engagement in kidney-related research.

**Methods:**

We conducted a thematic analysis of two online moderated discussions and an interactive online survey with open-text responses involving participants (i.e. stakeholders), with personal and/or professional experiences with Latinx patients with kidney diseases and their families/caregivers.

**Results:**

Among the eight stakeholders (Female:75%; Latinx ethnicity:88%), there were three physicians, one nurse, one patient with kidney disease who received a kidney transplant, one policy maker, one Doctor of Philosophy, and one executive director of a non-profit health organization. We identified five themes. The majority of themes and their respective subthemes (in parentheses) reflected barriers to engagement: Lack of personal relevance (unable to relate to research staff and marketing resources, and unclear benefit of research to self, family, and community); fear and vulnerability (immigration concerns, stigma with seeking care, skepticism of Western medicine); logistical and financial barriers (limited opportunities to enroll in clinical trials, out-of-pocket costs, transportation issues); and distrust and asymmetry of power (related to limited English proficiency or health literacy, and provider bias). The last theme centered on stimulating interest and establishing trust in the research process.

**Conclusions:**

To overcome barriers to engagement in kidney-related research and establish trust among potential Latinx research participants, stakeholders recommended employing cultural responsiveness and community-based strategies. These strategies can help identify local health priorities, enhance research recruitment and retention strategies, and establish partnerships that continue to elevate research endeavors aiming to enhance the health of Latinx individuals with kidney diseases.

**Supplementary Information:**

The online version contains supplementary material available at 10.1186/s12882-023-03128-y.

## Introduction

Over 60 million individuals comprise the Latinx (i.e., Hispanic, a non-gender-based term for Latino/Latina) population, making it the largest ethnic minority group in the United States (U.S.) [1]. This fast-growing group includes an admixture of individuals from varying races and ancestries, with diverse cultural beliefs, social determinants of health, and levels of acculturation [2–4]. The Latinx population is disproportionately burdened by social challenges contributing to persistent health disparities [4–7]. In terms of kidney-related disparities, U.S. Latinx individuals have 1.3 times the risk of developing kidney failure relative to White individuals [8], and are less likely to receive pre-kidney failure nephrology care, initiate kidney replacement therapy with home dialysis, or undergo transplantation.

Despite their disproportionate burden of kidney disease, the Latinx population is underrepresented in clinical research [9]. Garnering greater Latinx participation (and that of other socially marginalized groups) in clinical research may lead to stronger and more generalizable inferences, and deepen the understanding of therapeutic variation between racial and ethnic subgroups [10]. Previous research has identified participation barriers for Latinx patients; however, there may be barriers that are unique to Latinx patients living with chronic kidney disease (CKD) [11–17]. To increase Latinx participation in kidney research, it is critical to gather perspectives from Latinx patients with kidney disease, their families/caregivers and medical providers, kidney disparities researchers, and other relevant stakeholders. Herein, we discuss a unique opportunity to explore the perspectives of stakeholders with personal and professional experiences with Latinx patients with kidney diseases. In this thematic analysis, we aimed to describe stakeholders’ perspectives on engaging Latinx patients in kidney-related research.

## Methods

### Setting, participants, and data collection

We conducted a thematic analysis of data obtained from the transcripts of two online 2-hour moderated discussions and an interactive online survey with open text responses conducted by Travere Therapeutics (© 2023 Travere Therapeutics, Inc.), a biopharmaceutical company whose mission is to identify, develop, and deliver therapies to individuals with rare diseases [18]. The purpose of the internal research activity, organized by Travere, was to gain insights into engaging with Latinx communities and patients with kidney diseases, identify ways to communicate and educate about clinical trials and potential treatment options, and discuss best practices and opportunities to increase awareness of kidney diseases. The invited advisory board (herein referred to as stakeholders) were individuals with personal and/or professional experiences with Latinx patients with kidney diseases. Recruited stakeholders included representatives of patient advocacy organizations working with patients with kidney diseases, key opinion leaders involved in diversity, equity and inclusion activities, individuals working with healthcare organizations or academic institutions that provide care or outreach to Latinx communities, or were Latinx patients with a kidney disease. Purposive sampling was used to recruit participants, specifically, Travere representatives attempted to contact participants by phone calls and emails. Potential participants included individuals with a previous working relationship with Travere, and those identified via an internet search who met the recruitment criteria. Travere collected participant information including ethnicity, profession, state of residence, and sex. Travere’s engagement with stakeholders occurred via 1) an initial online 2-hour moderated discussion, 2) a two-week interactive survey via an online engagement platform, and 3) a closing online 2-hour moderated discussion (Supplemental Tables 1 and 2).

During the initial online moderated discussion, Travere representatives discussed the company’s goals to improve kidney disease outcomes and engagement with Latinx patients and discussed the planned activities for the investigation. An online engagement platform Within3 (© 2008–2023 Within3), was used for the 2-week interactive online survey with open-text responses. The questions for the online survey were developed by Travere based on literature review and gaps of knowledge perceived by the organization representatives and that of patient advocacy organizations working with racially and ethnically diverse patients. The questions were designed to elicit stakeholder perspectives on engaging Latinx patients in kidney-related research. There were two objectives of the online survey. The first was to understand the unique challenges and needs of the Latinx population with kidney disease, as well as the role of family. Communications discussing this objective occurred via Within3 over the first week of the activity. Second, stakeholders discussed issues related to Latinx participation in kidney-related research; this occurred during week two of the activity. At the beginning of each week, moderators posted questions to stakeholders related to the applicable objective, and stakeholders provided answers within the first three days of the week. All stakeholders were asked to answer every survey question. Moderators monitored the discussions and posed further questions to stimulate further dialogue. Following the two weeks of communications via Within3, stakeholders took part in a closing online moderated discussion where time was provided to review results, and consider potential solutions and collaborative opportunities.

Travere Therapeutics sponsored the activity for internal research purposes and sponsored publication costs but did not play a role in the thematic analysis or the final decision to publish this manuscript. All study protocols were granted an exemption from requiring ethics approval by the Colorado Multiple Institutional Review Board and participant informed consent requirements were waived. This study followed the Consolidated Criteria for Reporting Qualitative Research (COREQ) reporting guideline [19] (Supplemental Table 3).

### Analysis

Transcripts were imported into HyperRESEARCH (version 4.0.1 ResearchWare Inc. Randolph MA). Using thematic analysis, A.T. read transcripts and inductively identified preliminary concepts, and grouped similar concepts into initial themes and subthemes. These were reviewed and discussed with F.A. and L.C. A.T. coded the transcripts and identified patterns within the data. Consensus on themes and subthemes occurred following review of the thematic analysis (A.T., C.D., F.A., L.A., S.B.N.) The research team was composed of female scientists (with expertise in translational, patient-centered outcomes, and qualitative research), policy advocates, and physicians.

## Results

The eight stakeholders were comprised of three physicians (one nephrologist and two internal medicine physicians), one registered nurse, one patient with kidney disease who received a transplant, one policy decision-maker (Juris Doctor), one Doctor of Philosophy-trained health disparities researcher, and one executive director of a non-profit health organization. Seven out of the 8 stakeholders self-identified as Latinx at least once during the study; information about ethnicity was not available for one participant (Table 1).

**Table 1:** Participant characteristics, *n* = 8

Characteristic	n (%)
Female	6 (75)
Age: ≥ 18 years of age	8 (100)
Latinx ethnicity ^a^	7 (87.5)
**Region of Residence in the US**	
West	1 (12.5)
Midwest	0 (0)
Northeast	3 (37.5)
Southeast	1 (12.5)
Southwest	3 (37.5)
**Profession**^**b**^	
Nephrologist	1(12.5)
Internal medicine physician	2 (25)
Registered nurse	1(12.5)
Policymaker	1 (12.5)
Director of a Latinx health center	1 (12.5)
Disparities researcher	4 (50)
Marketing and Communications Director	1 (12.5)
Patient with kidney disease	1 (12.5)

^a^ Ethnicity not available for one participant ^b^Participants could have overlapping roles

**Fig. 1:**
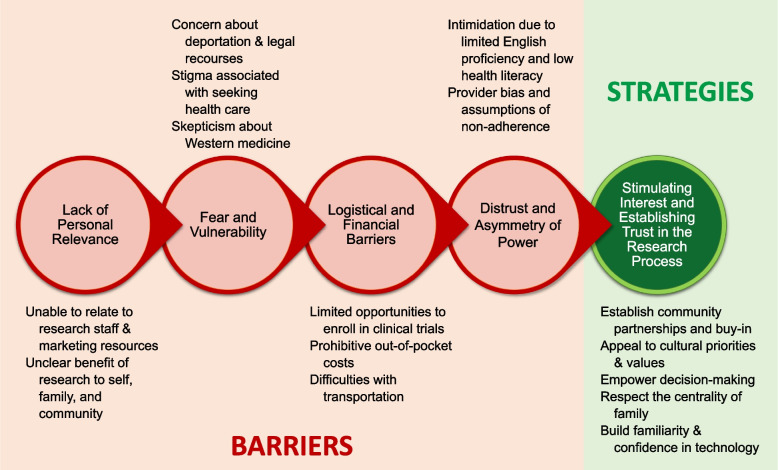
Thematic schema

We identified five themes: 1) lack of personal relevance, 2) fear and vulnerability, 3) logistical and financial barriers, 4) distrust and asymmetry of power, and 5) stimulating interest and establishing trust in the research process. The first four themes encompassed barriers that stakeholders perceived hindered Latinx participation in kidney-related research, whereas the fifth theme conveyed stakeholder-recommended strategies to overcome barriers. Respective subthemes are described in the following sections and in Fig. 1 with selected supporting quotations provided in Table 2.

**Table 2:** Selected supporting quotations

Theme	Selected illustrative quotations
**Lack of personal relevance**	
Unable to relate to research staff and marketing resources	“If the person in the ad does not appear relatable—you assume you must not qualify. You have to see something to believe it includes you.” “Opportunities to hear from clinical trial participants that look like them and have similar experiences in a culturally appropriate way might help address the lack of knowledge and create more receptivity.” “Clinical trials need to incorporate more Latino investigators, researchers and other staff so they can build trust, be Latino-centered and help increase participation of more Latinos.” “It starts from the inside out. What does the current staff look like? Is there any relatability? Not just race. And [it is] okay if there isn't but then you have to lean more into the communities where you want to help most and find people that the community trusts.”
Unclear benefit of research to self, family, and community	“I honestly [think] there is not enough information shared in the general public, let alone within Latino/Hispanic communities regarding Clinical trials.” “They are hesitant, mainly due to the fact of not knowing if they will receive a placebo or the real test.” “Talking about patients who are undocumented, and just thinking ahead, if you do get approval for your investigational product, sometimes it's even hard to get the medications through the patient assistance programs, because sometimes in some companies, if they can't document a legal status, a lot of times they won't qualify for their patient assistance programs to get them free medication.” “Parents and caregivers, may be hesitant to enroll their children in clinical trials if they perceive there will not be an immediate benefit to the child.” “Latinos would be very receptive to participating in clinical trials because they always want to help their families and communities.”
**Fear and vulnerability**	
Concern about deportation and legal recourses	“It needs to be explicitly stated that a patient's information will not be shared because many fear deportation when joining research.” “Patients who are undocumented may forego seeking medical care due to fear of deportation.” “[One patient] stopped coming to see me in the office because she was scared to leave her house for fear of being picked up and getting deported.”
Stigma associated with seeking healthcare	“The biggest challenge for Latinos/Hispanics is a strong stigma to seek care.” “In the community I serve, there is a lot of stigma shown as fear of "getting worse" and not being able to pay for follow up care.” “Beyond machismo, it's fear. People say 'If there's nothing wrong with me, why do I want to go and find out? My relative, so and so was fine until they went to the doctor. And now they have five different problems that they [did not] know before.' So it goes beyond this idea of machismo, but also just fear in general, that something will be found.” “If God wants me to have a problem, then I'll deal with it, then. I’m not going to deal with it until it's almost like destiny, but it's really more like fatalism.”
Skepticism about Western medicine	“Preference for non-Western medicine is also something that comes up as a reason (reliance on natural remedies).” “Of course, turning to friends and family members is also common among some Latinos seeking natural remedies from their most trusted circle of family and friends.”
**Logistical and financial barriers**	
Limited opportunities to enroll in clinical trials	“The primary care doctors … they're so overwhelmed. They're more worried about [getting] them to see the nephrologist… Do they have coverage? Can they get an appointment in time? A lot of times our communities are so underserved, that research is the icing on the cake. We're barely trying to get the plain vanilla cake to begin with.”
Prohibitive out-of-pocket costs	“For many Latinos/Hispanics it is not financially feasible for them to join a study… they maybe cannot take a day off from work to have blood work done or complete study visits, it may be too expensive for them to travel to a study visit (e.g. bus tokens, cost of gasoline, etc.), they may not have childcare and may need to pay for this out of pocket, etc. It is critical that these patients be well compensated.” “They would consider potential costs associated with the participation in the trial.” “Financial barriers for Latinos with low socio-economic status is a real impediment, in additional to the other social determinants of health.”
Difficulties with transportation	“Another common challenge (among older participants) is transportation. To circumvent this challenge we make in-home appointments or suggest public (yet private places) such as local public library study rooms. However, with clinical trials collecting biospecimen the library is not as feasible. We sometimes do hire Ubers or have institutional transportation vehicles bring patients to/from home.” “It's important for the sites to communicate about reimbursement of transportation costs (or provide vouchers)” “In terms of economic concerns we try to mitigate these by providing transportation (we have a designated staff member who picks up the patient and takes them back home after their visits), and by providing a stipend that is appropriate to compensate them for their time.”
**Distrust and asymmetry of power**	
Intimidation due to limited English proficiency and low health literacy	“Medical terms need to be broken down, but not to sound scarier.” “When you are [in] clinic and you see people looking through the forms, totally overwhelmed. A few bilingual staff members [are there] to help translate a bare minimum.” “I have seen patients go more out of their way to seek Spanish-speaking providers in areas where Spanish-speaking providers are less readily available.” “Addressing the lack of knowledge about clinical trials by developing bilingual educational materials in a manner that is culturally appropriate, especially for Latinos with lower educational attainment might eliminate this barrier.”
Provider bias and assumptions of non-adherence	“Latino patients reportedly have poor medication adherence, which is exacerbated by lower levels of health literacy, lower socio-economic status, as well as acculturation.” “Physicians' attitudes/bias about Hispanics not having the ability to follow through on the management protocols.”
**Stimulating interest and establishing trust in the research process**	
Establish community partnerships and buy-in	“You need to do things for the community like FREE Kidney Disease screenings, FREE cooking classes, create a cheat sheet (English/Spanish) that advises people what to look out for that makes them high risk, how to advocate for their own health.” “When participants see that the venue personnel communicate positively about recruiters they are more open to having dialogue with the recruiters.” “Community trust is key in research. I frequently see how study teams struggle to recruit participants because they want to send an email and get a positive response, but that's often not possible. It is important to incorporate community voices at the systems level to ensure people see us and we see them. Some common practices include establishing advisory boards, where researchers present their projects and consult with patients and present their findings, engaging in community programming, and developing translational research programming.” “Community centers themselves (e.g. adult education centers) have been able to provide information on other community resources (e.g. community clinics) that patients may leverage for health information and health care. Churches and religious centers have also been able to provide health information” “Securing "buy-in" with community clinic staff, and providers has greatly assisted my efforts in recruitment. Other venues such as public libraries, and local division of motor vehicles have been opened to us tabling and sharing information on available clinical trials.”
Appeal to cultural priorities and values	“Cultural targeting of information/education that speaks to music, food, family, and in some cases religion will pay dividends when it comes to getting potential participants interested in participating in clinical trials.” “We also make sure that our health communication pieces whether written or video is culturally tailored.” “Relevant to providing information or for recruitment efforts via morning show we use "Despierta America" to reach older audiences “It may be a multilayer approach including grassroots organizations, leaders, and community members. Conducting an assessment about how much they know about the topic and answering any general questions, following up with discussions and forums about their needs and fears, and facilitating channels to resolve systemic concerns.” “Another strategy is partnering with patient-oriented organizations to get the information to the patients. Again having research staff who is language/culture concordant is key.”
Empower decision-making	“Setting up a chat bot or frequently-asked-questions site with commonly asked questions. You could monitor and verify or flag posts from patient community. Provide multiple options to communicate (email/call/schedule [appointment]/text) Education and really understanding your diagnosis is powerful.” “When you were talking about videos and telenovelas and this and that, I thought that would be a great way to try to explain [the objective of the] clinical trial to a patient … we’re trying to get informed consent. Because a lot of times we basically give them a stack of papers and we go through it with them… Kind of goes over [their] head, and you wonder, does that really inform them or not?” “Patient advocacy groups are indeed a trusted source of health information. In my experience I have seen that membership into these groups are often suggested after diagnoses. However, once patients join, information shared within these groups is highly trusted among many members.” “Provide a list of ways they can be involved and allow them to volunteer to assist with patient recruitment.”
Respect the centrality of family	“The family wants to be involved and wants to participate in decision-making including the management of the illness. In my experience, when the family is involved in decision-making and understands the illness/management, the patient tends to be more adherent to medications because their families are involved and supporting them.” “Latinos consider family an important pillar to deal with problems (familism). In healthcare, it is important to follow a patient and family engagement approach that can discuss any issues and answers any questions from the patients and caregivers. This is particularly sensitive when patients are children, in which case parents are even more critical about how they care their children. I insist in the importance of providing culturally appropriate information in a timely manner. Talking with patients and caregivers, all the time and making them part of the patient's care is essential to ensure quality of care.” “Many Latinos seek advice and encouragement from extended family members. So there needs to be a concerted effort to engage family members in helping treat and manage chronic kidney disease. Women/mothers/wives especially play a big role in decision making.”
Build familiarity and confidence in technology	“Newly arrived immigrants have not had as in-depth access to technology and are thus disadvantaged in terms of health communications, or telehealth that leverage newer technologies.” “A critical programming need is telehealth/ mobile device/ electronic health record—patient portal training for medical providers and patients/participants.” “Availability of a mobile training team that can make in home visits would definitely facilitate patient comfort with digital health technology / telehealth devices they have been provided. This language-specific training should be made available to patients and caregivers.” “We do of course see that most older patients are likely to need assistance navigating the tablet.”

### Lack of personal relevance

#### *Unable to relate* to *research staff and marketing resources*

Stakeholders stated that Latinx patients may be reluctant to engage in research because they do not relate to those who appear in study advertisements. They suggested that patients “have to see something to believe it includes [them].” Stakeholders remarked there was a lack of Latino researchers, staff, and advocates who “look like the patient,” and that study communications were not always in the patient’s or family’s preferred language. “Take time to hire diverse staff who can help infuse cultural nuances that will be lost in translation.” Stakeholders believed these issues should be addressed since obtaining study information “from people [who] look like them, speak like them,” could help establish trust, and may thereby “create more receptivity” to participating in trials and other forms of clinical research.

#### Unclear benefit of research to self, family, and community

Stakeholders believed there were “not enough educational resources on the power of clinical trials.” The need for simple, culture- and language-concordant educational resources about the potential benefits of participation was advocated. One stakeholder (a kidney transplant recipient) remarked, “It wasn't until I got involved with [fundraising organization] that I really started to understand the importance of research and clinical trials.” Stakeholders were concerned that Latinx patients may not perceive a personal or direct benefit of engaging in trials or other forms of clinical research. One stakeholder (medical provider caring for patients with kidney disease) added: “Our patients are very receptive to participating because they can see how much more attention patients get in a clinical trial (this is very evident to the in-center hemodialysis patients who can see their peers participating in trials in the center).” Regarding the structure and processes, of clinical trials, not knowing whether patients would be placed in the intervention arm or control arm of a clinical trial contributes to hesitancy. Moreover, one stakeholder remarked that while investigational products were approved based on trial evidence, undocumented patients would not be able to access approved medications. As such, their participation may not be considered to directly benefit their own family and the larger community. Stakeholders emphasized informing patients of the “disparities and increased burden” of diseases affecting the Latinx community. “The average Latino/a may not know how diversity in clinical trials can improve patient health outcomes for themselves, their families and/or their communities.”

### Fear and vulnerability

#### Concern about deportation and legal recourses

Stakeholders believed fear of deportation was a major concern. They explained that undocumented patients were often reluctant to seek healthcare as they were terrified of being reported to authorities and that there was a “lack of assurance from clinical trial recruitment staff that immigration status [would be kept confidential].”

#### Stigma associated with seeking health care

Through previous personal and professional experiences with Latinx family members, friends, and patient individuals, stakeholders recognized a societal and cultural pressure to “always appear healthy,” and believed many Latinx individuals avoided being “diagnosed,” or “sick or weak,” and feared that receiving a diagnosis would threaten their source of income. Stakeholders described how some individuals feared that by seeking care, their health would deteriorate, and they would not be able to afford follow-up. Stakeholders believed the “strong stigma” attached with seeking care was inadvertently transferred to the context of trial participation.

#### Skepticism about western medicine

Stakeholders suggested that Latinx individuals may be averse to enrolling in trials evaluating pharmacological interventions as patients had a “preference for non-western medicine” and wanted to rely on natural remedies.

### Logistical and financial barriers

#### Limited opportunities to enroll in clinical trials

Stakeholders believed that many medical providers serving Latinx communities felt “overwhelmed” having to address social challenges experienced by their patients and were more focused on ensuring appropriate medical care and follow-up over enrolling Latinx patients in research. These logistical barriers hinder Latinx patients’ ability to even enter clinical research studies.

#### Prohibitive out-of-pocket costs

Participating in a trial was expected to impose a financial burden on Latinx individuals. Stakeholders were concerned that the costs of taking time off work and childcare would be prohibitive for Latinx individuals of low-socioeconomic status who often were uninsured. Stakeholders advocated for compensation to address the financial obstacles of study participation. “Ramping up the financial incentives for low-income potential enrollees may also address part of the obstacle to participating.”

#### Difficulties with transportation

The cost and challenges of arranging transportation were identified as a major impediment to participating in trials. “It may be too expensive for them to travel to a study visit.” Stakeholders suggested arranging transportation or providing vouchers, scheduling “in-home appointments” and using easily accessible community venues to address transportation-related barriers. One stakeholder commented, “I can't stress enough how important the transportation piece is. The transportation is not as important for [patients receiving in-center hemodialysis] because they're already coming to the dialysis center, but for the non-dialysis, chronic kidney disease patients, sometimes they have to travel 35–40 miles.”

### Distrust and asymmetry of power

#### Intimidation due to limited English proficiency and low health literacy

Stakeholders remarked that Latinx individuals may feel “reluctant to admit when they don’t understand.” When having to fill out forms in clinical settings, for example, bilingual staff members may be able to “translate a bare minimum,” but even in a group setting, patients may feel “intimidated to admit they don't understand.” In the context of clinical care, Latinx individuals may “play down their illness, pain,” which could inadvertently delay referrals to specialists or relevant clinical trials for an underlying disease process.

#### Provider bias and assumptions of non-adherence

Some stakeholders believed that physicians may not discuss research opportunities with Latinx patients as they assumed that Latinx patients had “poor medical adherence” exacerbated by poor health literacy and low socioeconomic status. This biased perspective may make providers less inclined to enroll Latinx patients in studies.

### Stimulating interest and establishing trust in the research process

#### Establish community partnerships and buy-in

To foster interest in trials, stakeholders suggested that research teams harness community events and settings, and that they deliver information through trusted community members. “You have to lean more into the communities where you want to help most and find people that the community trusts.” They explained that taking action to help the community (e.g. free screening for kidney disease) could pique the interest in research among Latinx patients and families. Prioritizing visibility of community-centeredness was deemed critical to establish trust.

#### Appeal to cultural priorities and values

“Cultural targeting” of information and education that resonated with Latinx communities was underlined as a vital strategy to promote engagement. This could include the integration of community priorities such as family, food, music, and religion, and sharing information via news and media channels that were familiar and trusted by the target community. Specifically, framing participation in research studies as an opportunity to “help their families and communities” may further resonate with Latinx individuals. Stakeholders also recommended obtaining input early on from community members or “Latino organizations” to help ensure cultural appropriateness in research designs and outreach strategies.

#### Empower decision-making

Stakeholders suggested that Latinx patients and families should “feel completely comfortable with their decision to consent.” They believed it was important to present information in a clear and engaging manner (e.g. using videos rather than “a stack of papers”), provide sufficient time for decision-making, and opportunities to ask questions using multiple platforms, including by email, phone call, or during face-to-face appointments.

#### Respect the centrality of family

Stakeholders raised the concept of “*familismo*” and explained that for many Latinx individuals, “family comes before self” and that the family “took on the burden” of the patient’s illness by providing emotional and social support and participating in decision-making. Latinx patients would “seek advice and encouragement from extended family members.” Family members were recognized to have the “power to influence the perception and behavior of the patient.” Family members also had a role in “bridging” cultural aspects in managing the illness, for example, “younger generations being information brokers for older generations.” Moreover, family support could also encourage adherence to treatment and thereby trial protocols.

#### Build familiarity and confidence in technology

The increasing use of technology in trial recruitment and follow-up was acknowledged and thus stakeholders suggested providing access to, and training, in the use of technology. For example, they suggested that a mobile training team could conduct home visits to conduct “language-specific training” with patients in the use of health technology and telehealth devices (e.g. blood pressure monitors).

## Discussion

In this thematic analysis we identified five themes relating to Latinx engagement in clinical research. The first four themes conveyed barriers to engagement: lack of personal relevance, fear and vulnerability, financial and logistical barriers, and distrust and/or asymmetry of power. We also described stakeholders’ recommendations to overcome these barriers, centering around a fifth theme of stimulating interest and establishing trust in the research process. A unique aspect of our study is that we provide perspectives from stakeholders with personal and professional experience with Latinx patients with kidney disease. Previous studies exploring barriers and facilitators to research participation among Latinx patients have largely involved persons with non-kidney-related conditions [11, 20–23]. Previously, one international qualitative study explored patient/caregiver perspectives regarding their involvement in CKD research, however, participants were English-speaking, mainly of White race, and did not include Latinx participants [24].

Among strategies to build trust and enhance Latinx participation in clinical research, similar to previous recommendations, stakeholders advocated for cultural responsiveness (i.e. recognizing and respecting cultural differences and attempting to accommodate those differences) [20, 25]; this could include asking Latinx participants about their language preferences (and providing appropriate language access services, if needed), hiring research staff who are bilingual and bicultural, and/or partnering with trusted community members that may provide relevant insight into the community’s needs, values and priorities [7, 21, 26, 27]. More recently, culture- and language- concordant peer-navigators interventions have been explored as a means to improve patient-centered and clinical outcomes in kidney disease [28]. Similar culturally responsive peer navigator systems could be adapted for study recruitment and implementation purposes. Consistent with previous literature, stakeholders underscored the influence of *familismo* (familism), the “strong sense of importance and connectedness in family relationships and obligations” [29]. Family members may act as protective gatekeepers or information brokers, ultimately influencing health beliefs and behaviors such as medication adherence, and lifestyle changes [21, 29, 30]. Family members may also impact research participants’ willingness to engage in research studies, thus family/caregiver involvement should be encouraged early-on.

To further stimulate interest and trust in the research process, it is critical to involve the public (i.e. the community) in the design, conduct, and dissemination of research. Several research approaches and frameworks have been developed to facilitate partnership and collaboration between researchers and stakeholders including community-based participatory research (CBPR), patient and public involvement (PPI), the Patient-Centered Outcomes Research Institute (PCORI) dissemination and implementation framework, and the Public involvement in research: values and principles Framework (INVOLVE) [21, 31–34]. CBPR, specifically, is rooted in the “active involvement of community members, organization representatives and researchers in all aspects of the research process,” wherein partners contribute their expertise to improve the understanding of the social, structural, or physical inequities impacting the community [35]. Stakeholders expressed that research teams should at the onset, plan for adequate time and financial resources to establish and maintain community partnerships [21]. To sustain partnerships, stakeholders also recommended that research teams maintain personal communication with community partners and demonstrate reciprocity (by volunteering or participating in community events); this is consistent with previous recommendations in the literature [21, 36]. Importantly, it is well documented that CBPR has informed many successful interventions among Latinx research participants with varying chronic diseases [21, 29, 36–40].

Consistent with previous studies, our findings highlight several psychosocial and structural barriers experienced by Latinx patients. Psychosocial barriers include mistrust in the research process, fear of discrimination, and confidentiality, particularly as it relates to immigration status [11–14, 21]. Strategies to help overcome these barriers include providing Latinx patients and their family/caregivers with information describing how the research team will maintain confidentiality, and establishing partnerships with community-based organizations to increase public trust in research processes [41]. Structural barriers include financial concerns, time constraints due to work or caregiving responsibilities, lack of transportation, and communication issues [7, 12, 15, 16]. Many of these structural barriers are remediable. Research teams may provide financial compensation for time off work, childcare needs, etc. Additional compensation to cover travel expenses could include vouchers, bus tokens, or independent/private vehicles arranged by the research team. Other considerations to mitigate transportation or participant-scheduling issues include choosing sites for data collection and interviews near participants’ home or workplace, easily accessible public locations such as libraries, or arranging in-home visits; this might also allow scheduling appointments outside of working hours. With the recent uptake of telehealth use and advances including smartphones, investigators could incorporate virtual video appointments for baseline and follow-up survey/data collection (in addition to the standard practice of telephone follow-ups), or even invest in virtual peer navigator systems to assist participants. Leveraging mobile internet and smartphone use is particularly relevant since most Latinx individuals (94%) report using a mobile device to access the internet [42].

### Limitations

This thematic analysis has several limitations. Questions posed in the online moderated discussions and the interactive online-survey were tailored for internal investigational purposes by Travere Therapeutics, participation was limited to eight stakeholders of which only one was a patient with kidney disease [24], and important participant characteristics such as self-reported race, gender, sexual orientation, or preferred language were not queried. Social desirability bias may have led to censorship of negative opinions about Latinx engagement in research, particularly since stakeholders were not blind to other participants’ identification or credentials. Further, we were unable to further probe study participants regarding their responses. Nevertheless, the methods provided a rich depiction of the stakeholders’ perspectives on the barriers hindering Latinx engagement in kidney-related research, and strategies to overcome them. Future studies should incorporate more patient perspectives on research participation and should include Latinx patients of varying stages of CKD; this will help researchers address multi-level social risk factors and other challenges.

## Conclusion

Numerous challenges uniquely impacting Latinx patients with kidney disease limit their ability to participate in clinical research. As a large, rapidly-growing and heterogenous population, it becomes near impossible to delineate all possible strategies to enhance engagement among Latinx individuals or to know which strategies are more relevant for a specific community. Instead, the leading approach is to incorporate community engagement and cultural responsiveness at the onset. Doing so will help investigators understand the health priorities of the community, build trust, enhance research recruitment and retention strategies, and establish long-lasting partnerships that will continue to guide and elevate research endeavors aiming to enhance the health of all Latinx communities with kidney disease.

## Supplementary Information

**Additional file 1: Supplemental Table 1.** Meeting Outline for Introductory and Closing Webinars.** Supplemental Table 2.** Virtual engagement platform questions.** Supplemental Table 3.** Consolidated Criteria for reporting qualitative studies (COREQ): 32-Item Checklist.

## Data Availability

All data generated or analysed during this study are included in this published article [and its supplementary information files].

## Abbreviations

CKD: Chronic kidney disease
COREQ: Consolidated Criteria for Reporting Qualitative Research
CBPR: Community-based participatory research
PPI: Patient and public involvement
PCORI: Patient-Centered Outcomes Research Institute
INVOLVE: Public involvement in research: values and principles Framework
