# Supplementary material for: Qualitative analysis of stakeholder perspectives on engaging Latinx patients in kidney-related research
Source: BMC Nephrol. 2023 Mar 30;24:79. doi: 10.1186/s12882-023-03128-y (PMC10061843; doi:10.1186/s12882-023-03128-y)
Supplement: Supplementary file 1 — Additional file 1: Supplemental Table 1. Meeting Outline for Introductory and Closing Webinars. Supplemental Table 2. Virtual engagement platform questions. Supplemental Table 3. Consolidated Criteria for reporting qualitative studies (COREQ): 32-Item Checklist. [file 12882_2023_3128_MOESM1_ESM.docx]

| **Supplemental Table 1.** Meeting Outline for Introductory and Closing Webinars | |
| --- | --- |
| Webinar Kick-off | Agenda:   1. Welcome and Introductions 2. Travere’s Commitment to Rare Disease 3. Review of Kidney Diseases and Prevalence in the Latino/Hispanic Community 4. Within3 Platform and Engagement 5. Next Steps and Q&A |
| Closing Webinar | Questions   1. **If they had the support services, and if people knew about clinical trials, would they be willing to participate?** 2. **Is there anything that you would like to add?** 3. **How can we prioritize engagement activities?** 4. **What are your thoughts on incorporating digital resources and telehealth into clinical trials?** 5. **Is there anything that you would like to add?** 6. **Did we miss anything?** 7. **Anything you’ve thought of after the discussion board was over?** 8. **What do you think is the most important takeaway for you or us from this advisory board?** |

Questions in **BOLD** relate to patient engagement in research

|  | |  |
| --- | --- | --- |
| **Supplemental Table 2.** Virtual engagement platform questions. | | |
| Within3  Platform  Questions,  Week 1 | Topic: Challenges, Resources, Role of Family Members   1. Based on your experience as a healthcare provider or an individual who works with the Latino/Hispanic patient/family caregiver, what are some of the major challenges the Latino/Hispanic patient/community face in receiving medical care to get a proper diagnosis and treatment?    1. Which of these would you prioritize as the highest priority obstacles?    2. What level of confidence/comfort would you say that Latinx/Hispanic patients have in the medical setting?    3. How often are Spanish-translated materials available? Is there an issue with different dialects of Spanish? 2. In the Latino/Hispanic community, is there a preference in being seen or treated by a Latino/Hispanic doctor versus a doctor of another ethnicity? 3. Upon first diagnosis of a rare kidney disease or other chronic diseases, what information, support and resources are patients looking for? What information/resources are provided to patients/caregivers? Are these resources typically in Spanish?    1. What is the emphasis on family members/generational opinion, knowledge or outreach; how much are family members’ opinions and influence valued in the disease management of the patient? 4. Where do patients and caregivers turn to when they need to get trusted/credible health information?    1. Who are the advocates in this space?    2. How do generational differences impact how individuals get their health information?    3. What support groups or communities are well-received or trusted?    4. How can one best deliver information to communicate with this community? 5. What is crucial to know about the role of other family members in the health/disease management of the patient? 6. What is crucial to know about the role of the caregiver for pediatric patients? 7. How does immigration status affect the Latino/Hispanic patient to get appropriate medical care? | |
| Within3  Platform  Questions,  Week 2 | **Topic: Clinical Trials, Patient Engagement, Support, and Resources**   1. **What is the overall opinion and receptivity of Latino/Hispanic patients when it comes to participating in clinical trials?** 2. **What is the overall opinion and receptivity of Latino/Hispanic caregivers when it comes to enrolling children/pediatric patients in clinical trials?** 3. **What obstacles need to be overcome for patients to participate in or have access to clinical trials? Consider socioeconomic, political, cultural concerns or other obstacles.**    1. **What is most impactful for this community to know about clinical trials?**    2. **What is a motivator for this community to join clinical trials?**    3. **How can we get the community to view the value of clinical trials?**    4. **What are some misconceptions? How can we tackle them?** 4. **How should clinical trial site staff engage the community for clinical study recruitment? What programs, education and resources should they provide to the patient/family during engagement?**    1. **If anyone has experience with clinical trials, is there anything we can provide the clinical trial site staff to best engage with the community that has been successful?**    2. **What is important to remember with this specific population? Best practices?**    3. **Are there any words/visuals/red flags to avoid?**    4. **Is there outreach or tactics that are generally received poorly?** 5. **How should a company like Travere Therapeutics work with Latino/Hispanic organizations or healthcare professionals to assist in patient recruitment of clinical trials in kidney diseases?** 6. If language was not an issue, what kind of support programs would be valuable to the patient and the family regarding management of chronic disease like kidney disease? | |
| Questions in **BOLD** relate to patient engagement in research | | |

| **Supplemental Table 3.** Consolidated Criteria for reporting qualitative studies (COREQ): 32-Item Checklist | | |
| --- | --- | --- |
| **No. Item** | **Guide questions/description** | **How manuscript adheres to the reporting standards** |
| **Domain 1: Research team and reflexivity** | | |
| **Personal Characteristics** | | |
| 1. Interviewer/facilitator | Which author/s conducted the interview or focus group? | N/A (Conducted by Travere) |
| 2. Credentials | What were the researcher’s credentials? | FA: MD, MHS, MPH; CD: MD; SBN: MD, MPH, PhD; AJ: PhD; LC: MD |
| 3. Occupation | What was their occupation at the time of the study? | FA, CD, SBN, LC: Physician-Scientist  AJ: Principal Research Fellow |
| 4. Gender | Was the researcher male or female? | All female |
| 5. Experience and training | What experience or training did the researcher have? Relationship with participants? | The research team was composed of scientists (with expertise in translational, patient-centered outcomes, and qualitative research), policy advocates, and physicians. |
| **Relationships with participants** | | |
| 6. Relationship established | Was a relationship established prior to study commencement? | No |
| 7. Participant knowledge of the interviewer | What did the participants know about the researcher? | N/A |
| 8. Interviewer characteristics | What characteristics were reported about the interviewer/facilitator? | N/A |
| **Domain 2: Study Design** | | |
| **Theoretical Framework** | | |
| 9. Methodological orientation and Theory | What methodological orientation was stated to underpin the study? | Study devised to elicit perspectives on engaging Latinx patients with kidney-related research (based on literature review, perceived organization gaps) |
| Participant selection | | |
| 10. Sampling | How were participants selected? | Invited as stakeholder if the representative of patient advocacy organizations, key leader involved in diversity, equity, and inclusion activities, working with health care organizations or academic institutions providing care or outreach to Latinx patients or patients with kidney disease |
| 11. Method of approach | How were participants approached? | By email, phone |
| 12. Sample size | How many participants were in the study? | 8 |
| 13. Non-participation | How many people refused to participate or dropped out? | Not known |
| 14. Setting of data collection | Where was the data collected? | Online (Zoom, Within3) |
| 15. Presence of non-participants | Was anyone else present besides the participants and researchers? | Travere representatives |
| 16. Description of sample | What are the important characteristics of the sample? | Female:75%; Latinx ethnicity: 88% |
| **Data Collection** | | |
| 17. Interview guide | Were questions, prompts, guides provided by the authors? Was it pilot tested? | Yes- questions were provided. Questions were not pilot tested |
| 18. Repeat interviews | Were repeat interviews carried out? | No |
| 19. Audio/visual recording | Did the research use audio or visual recording to collect the data? | Both |
| 20. Field notes | Were field notes made during and/or after the interview or focus group? | No |
| 21. Duration | What was the duration of the interviews or focus group? | Webinars: two hours. Online surveys: up to one week per topic |
| 22. Data saturation | Was data saturation discussed? | No |
| 23. Transcripts returned | Were transcripts returned to participants for comment and/or correction? | No |
| **Domain 3: analysis and findings** | | |
| 24. Number of data coders | How many data coders coded the data? | 1 |
| 25. Description of the coding tree | Did authors provide a description of the coding tree? | No |
| 26. Derivation of themes | Were themes identified in advance or derived from the data? | Derived from data |
| 27. Software | What software, if applicable, was used to manage the data? | HyperRESEARCH (version 4.0.1 ResearchWare Inc. Randolph MA) |
| 28. Participant checking | Did participants provide feedback on the findings? | Yes |
| **Reporting** | | |
| 29. Quotations presented | Were participant quotations presented to illustrate the themes/ findings? Was each quotation identified? | Yes – see tables |
| 30. Data and findings consistent | Was there consistency between the data presented and the findings? | Yes – see results |
| 31. Clarity of major themes | Were major themes clearly presented in the findings? | Yes – see results |
| 32. Clarity of minor themes | Is there a description of diverse cases or discussion of minor themes? | Yes – see results |

Developed from: Tong A, Sainsbury P, Craig J. Consolidated criteria for reporting qualitative research (COREQ): a 32-item checklist for interviews and focus groups. *International Journal for Quality in Health Care*. 2007. Volume 19, Number 6: pp. 349 – 357
